# Supplementary material for: Patterns of Recurrence after Neoadjuvant Therapy in Early Breast Cancer, according to the Residual Cancer Burden Index and Reductions in Neoadjuvant Treatment Intensity
Source: Cancers (Basel). 2021 May 20;13(10):2492. doi: 10.3390/cancers13102492 (PMC8161089; doi:10.3390/cancers13102492)
Supplement: Supplementary file 1 [file cancers-13-02492-s001.zip › cancers-1161679-supplementary.pdf]

cancers

MDPI

Patterns of Recurrence after Neoadjuvant Therapy in Early Breast Cancer, according to the Residual Cancer Burden Index and Reductions in Neoadjuvant Treatment Intensity

Christoph Suppan, Florian Posch, Hannah Deborah Mueller, Nina Mischitz, Daniel Steiner, Eva Valentina Klocker, Lisa Setaffy, Ute Bargfrieder, Robert Hammer, Hubert Hauser, Philipp J. Jost, Nadia Dandachi, Sigurd Lax and Marija Balic

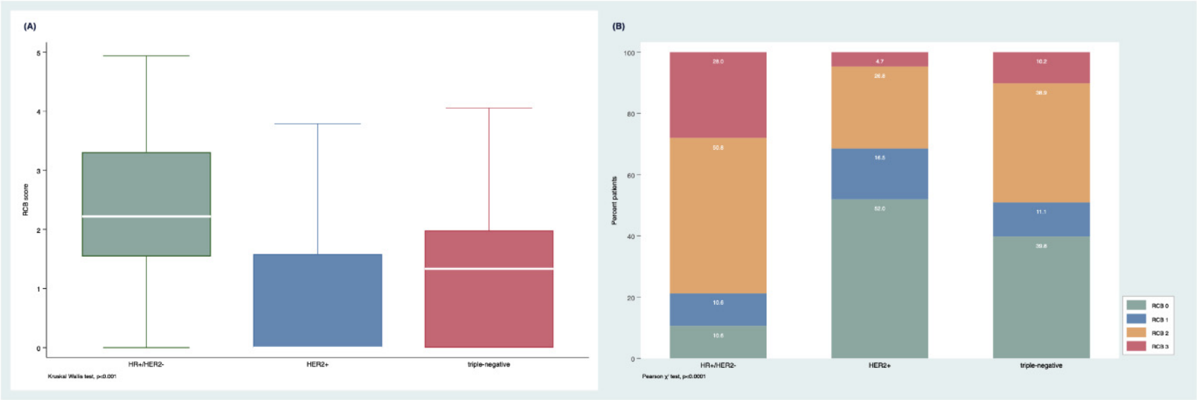

Figure S1. Association between RCB score (**A**) and RCB class (**B**) with breast cancer subtypes.
